# Identification and bioactive potential of marine microorganisms from selected Florida coastal areas

**DOI:** 10.1002/mbo3.448

**Published:** 2017-01-26

**Authors:** Anna Christensen, Glenroy D. A. Martin

**Affiliations:** ^1^ College of Medicine University of South Alabama Mobile AL USA; ^2^ Department of Life and Physical Sciences Fisk University Nashville TN USA

**Keywords:** Actinobacteria, antimicrobial activity, *Halichondria panicea*, microbial diversity, molecular taxonomy, Proteobacteria

## Abstract

The ocean, with its rich untapped chemical biodiversity, continues to serve as a source of potentially new therapeutic agents. The evaluation of the diversity of cultivable microorganisms from the marine sponge *Halichondria panicea* and ocean sediment samples were examined and their potential as sources of antimicrobial and antiproliferative agents were investigated. The marine sponge and sediments were collected at different depths (0.9–6 meters) and locations in Florida, including Florida Keys, Port St. Joe in Pensacola, Pensacola Bay, Pensacola Beach, and Fort Pickens. Twenty‐one cultivatable isolates were grouped according to their morphology and identified using 16S rRNA molecular taxonomy. The bacterial community identified consisted of members belonging to the Actinobacteria, Bacteroidetes, Proteobacteria (Alpha‐ and Gamma‐classes) and Firmicutes phylogeny. Seven of the microbes exhibited mild to significant cytotoxic activities against five microbial indicators but no significant cytotoxic activities were observed against the pancreatic (PANC‐1) nor the multidrug‐resistant ovarian cancer cell lines (NCI/ADR). This work reaffirms the phyla Actinobacteria and Proteobacteria as sources of potential bioactive natural product candidates for drug discovery and development.

## 1 Introduction

The rich biodiversity of the ocean continues to afford marine natural products that serve as a framework for the development of new therapeutic agents. To date, there are a number of success stories of marine‐inspired approved drugs along with more at different stages of Phase I–III clinical trials (Becker & Terlau, 2008; Fenical et al., 2009; Gerwick & Fenner, 2013; Huyck, Gradishar, Manuguid, & Kirkpatrick, 2011). The majority of these bioactive chemical entities have been isolated from marine invertebrates, such as molluscs, bryozoans, tunicates and sponges, as well as from marine bacteria and cyanobacteria (Mehbub, Lei, Franco, & Zhang, 2014).

**Figure 1:**
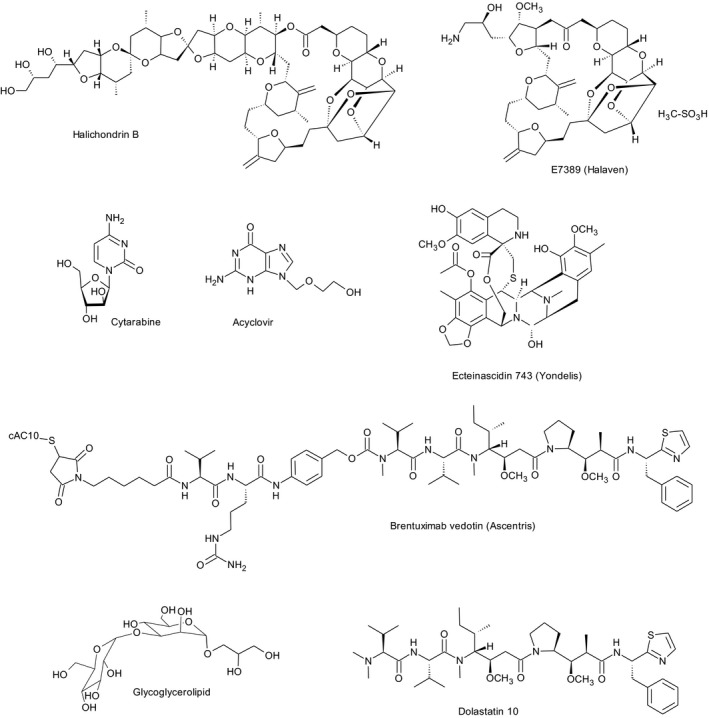
Marine natural products and their clinical derivatives

Marine sponges are widely distributed across the various depths of the ocean floor with a few being present in freshwater environs (Perdicaris, 2013; Thomas, Kavlekar, & LokaBharathi, 2010). As filter feeders (Mehbub et al., 2014), they not only consume microorganisms and organic particles but also produce potent chemicals as a defense mechanism against competitors, predators, and infectious microorganisms (Gomes Filho et al., 2015). These chemical entities have proven to be important to the pharmaceutical industry due to their antitumor, antimicrobial, antiviral, and cytotoxic properties (Jensen & Fenical, 1994; Wang, 2006). Ara‐A (acyclovir) and Ara‐C (cytosar‐U, cytarabine) are two such examples of pharmaceutical drugs that are obtained from the marine sponge *Tethya crypta* (Figure 1). The former is used as an antiviral drug for the treatment of herpes infections (Murti & Agrawal, 2010) while the latter serves as an anticancer drug for leukemia and non‐Hodgkin's lymphoma (Md, Fareed, Ansari, & Khan, 2012). Another example is E7398 (Halaven^®^) which is derived from Halichondrin B, an isolate of the Japan Sea sponge *Halichondria okadai*. It exhibits strong activity against a range of different tumors, especially breast carcinoma (Towle et al., 2012).

Recent studies have confirmed that marine microbes associated with macroorganisms are actually the true producers for most of the reported bioactive chemical entities. Those of the phyla Proteobacteria and Actinobacteria have the capacity to yield novel bioactive secondary metabolites (Blunt, Munro, Copp, Keyzers, & Prinsep, 2012; Dupont et al., 2014; Gerwick & Moore, 2012; Mayer et al., 2010). Ecteinascidin 743 (Yondelis^®^), for example, that was originally isolated from the tunicate *Ecteinascidia turbinata* is now purportedly produced by the tunicate's uncultured symbiotic bacterium *Endoecteinascidia frumentensis*. It is used for treatment of ovarian cancer and soft tissue sarcoma (Martínez et al., 2005). Likewise, the isolation of dolastatin 10 from the sea hare *Dolabella auricularia* has been shown to be biosynthesized by the marine cyanobacterium *Symploca* sp. dolastatin 10's conjugated analog, brentuximab vendotin (Ascentris^®^), is administered to patients with Hodgkin's lymphoma and systemic anaplastic large‐cell lymphoma (Figure 1). Given the complex structural nature of Halichondrin B, it too has been suggested to be of microbial origin (Gerwick & Fenner, 2013).

The utilization of marine microbes as the primary source of bioactive natural products is important in addressing the supply issue faced during the development of potentially new pharmaceuticals from macroorganisms (Leal et al., 2014). Cultivation of these microbes in the laboratory, however, can be challenging (Kaeberlein, 2002). This is partly due to the inability of growth media to adequately mimic natural environments (Pham & Kim, 2012). In addition, many species are not viable, or may require long growth periods before they become visible. Previous studies have successfully cultured novel microbes using encapsulation procedures, diffusion growth chambers, casein and microorganism specific agars, and media enriched with selective antibiotics (Zengler et al., 2002). The incorporation of seawater with dilute agars and the development of other specialized growth media have been successful in helping to get fastidious microorganisms into culture (Jensen, Gontang, Mafnas, Mincer, & Fenical, 2005).

In our continued efforts to identify new marine microorganisms as potential sources of novel bioactive natural products, the microbial communities associated with marine sediments and the sponge *Halichrondria panicea* from diverse and previously unexplored areas located off the Florida coast were examined. Previous studies of *H. panicea* have resulted in the isolation of a *Microbacterium* sp. and subsequent anticancer glycoglycerolipid (1‐*O*‐acyl‐3‐[*R*‐glucopyranosyl‐(1‐3)(6‐*O*‐acyl‐*R*‐mannopyranosyl)]‐glycerol) in this organism collected from Adriatic coast in Rovinj, Croatia (Joseph & Nair, 2013; Wicke et al., 2000). The outcome of these efforts will serve as the basis for the future isolation and testing of the bioactive chemical constituents from the microbial extracts.

## 2 Materials and Methods

### 2.1 Sample collection and processing

**Figure 2:**
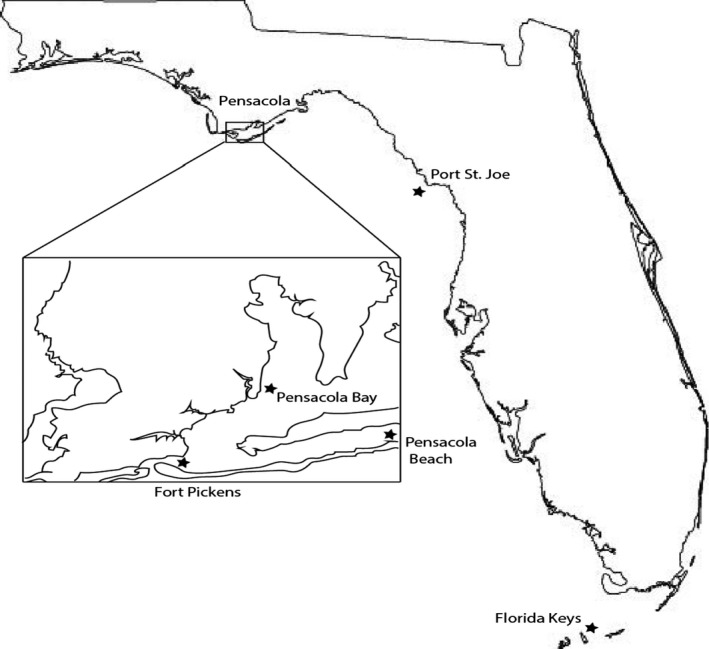
Florida coastal region sample collection sites utilized in this study

Thirty‐four samples were collected from diverse Florida marine areas (Figure 2). Sediments from depths ranging from 0.9 to 1.8 m were collected by snorkeling and those from depths of 3.7–6 m were collected by scuba divers. The upper 5 cm of sediment were collected into 50‐ml Eppendorf tubes (50% sediment and 50% sea water) to mimic aerobic conditions. The samples collected were either frozen or stored at 4°C and were processed under sterile conditions on the same day or the next. Five sediment samples and a clump of the green‐grey sponge *Halichondria panicea* (Hooper & van Soest, 2002; Little, 1963) were collected from Port St. Joe at depths of 0.9–1.8 m in July 2007. Five other sediment samples were collected on April 2009 from the Florida Keys along the shallow shoreline of Grassy Key Quarry (24º44′56ʺN, 80º58ʹ43ʺW) and Missouri Key (24º40′40ʺN, 81º14ʹ10ʺW). Seven sediment samples were collected around the Emanuel Point shipwreck (30°20′24″N, 87°13′48″W) in the Pensacola Bay on July 2009 in 3.7 m of water, and 18 samples were collected on September 2009 from the sandy Pensacola Beach and Fort Pickens (30°19′50″N, 87°17′03″W) areas in depths of 4.3–6 m of water. Under sterile conditions in a biological safety cabinet, the *H. panicea* sponge was rinsed with sterile water before a cotton swab was used to transfer microbes from a cut cross‐section onto each of the selected agar media.

The sediment samples were processed using previously reported methods (Gontang, Fenical, & Jensen, 2007; Hameş‐Kocabaş & Uzel, 2012; Öner et al., 2014) in order to facilitate the growth of spore forming Gram‐positive bacteria which are associated with the production of bioactive chemical entities. They were processed using one of four methods: *Method 1* (wet/stamp), under sterile conditions, 90% of the sea water was poured off, and a sterile cotton swab used to lightly stamp the sediments onto selected agar media; *Method 2* (dry/dilute), a small spatula of sediment (c 0.5 g) was transferred to a sterile glass Petri dish, allowed to dry overnight, and then diluted with sterile sea water (5 ml). This was stamped onto the agar surface using a sterile cotton swab; *Method 3* (dilute/heat), dried sediment was diluted with sterile seawater (1:4 dilution) and heated to 55°C for 6 min (Jensen et al., 2005), and the resulting suspension stamped onto the agar with a cotton swab; *Method 4* (freeze/dilute), frozen sediment was thawed, diluted with sterile seawater (1:4 dilution), and the diluted sample plated onto agar using methods 2 and 3. The processed samples were inoculated onto selected media and the microbes allowed to grow at 20°C for 2–6 weeks.

### 2.2 Isolation media of culturable isolates

The isolation media consisted of the following: nutrient agar (23 g) with either seawater or distilled water (1 L); marine agar (55.1 g) with either seawater or distilled water (1 L); actinomycetes agar (22 g) with distilled water (1 L); M1, agar (18 g), starch (10 g), yeast extract (4 g), peptone (2 g), rifampin (5 μg/ml), and natural seawater (1 L) (Gontang et al., 2007); M2 (10% M1), agar (18 g), starch (1 g), yeast extract (0.4 g), peptone (0.2 g), and natural seawater (1 L); M3, agar (18 g), starch (2.5 g), yeast extract (1 g), peptone (0.5 g), glycerophosphate (0.2 g), natural seawater (750 ml), and DI water (250 ml); M4 (100% seawater agar), agar (18 g), and natural seawater (1 L); ISP2 (M987), agar (20.0 g), yeast extract (4.0 g), malt extract (10.0 g), dextrose (4.0 g), and distilled water (1 L). The media were obtained from BD, Fisher Scientific, MP Biomedical and Sigma Aldrich.

### 2.3 Cultivation, extraction, and bioactivity testing

Single pure colonies from each of the selected microorganism were grown in liquid media (1 L) similar to the agar media on which it was originally isolated. The flasks were shaken at 200 rpm at 27°C and the fermentation allowed to proceed for 5 days. The fermentation broth was pooled and the microbial cells were separated from the broth via vacuum filtration. The broth was extracted with a 50:50 methanol:methylene chloride (2 × 500 ml), and the cells were extracted in 50:50 methanol:methylene chloride (500 ml). The organic solution from the broth was removed *in vacuo* and the aqueous portion re‐extracted with ethyl acetate (2 × 500 ml). The organic solutions from the broth and cells were dried separately with anhydrous sodium sulfate, filtered, and concentrated *in vacuo* to afford crude extracts. Other liquid media fermentations (2 L) were scaled up accordingly.

The extracts were tested for cytotoxic activity against the microorganisms *Candida albicans* ATCC 44506*, Saccharomyces cerevisiae*, the Gram‐positive bacterium *Staphylococcus aureus* ATCC 29213, the Gram‐positive bacterium *Bacillus subtilis* ATCC 6633, and the Gram‐negative bacterium *Pseudomonas aeruginosa* ATCC 27853 using standard disk diffusion assays (Harley, Prescott, Klein, & Harley, 2005; Larkin, 1982). Each crude extract (250 μg solubilized in ethanol) was allowed to dry on a 6‐mm disk before being applied to the agar surface inoculated with the test organisms. Zones of inhibition were recorded after 48 hr of co‐incubation at 37°C. A test microorganism was considered resistance to an extract if microbial growth was present around the disk. Control compounds included nystatin (100 U) for the yeast, and gentamicin (10 μg) and carbenicillin (100 μg) for the bacteria.

Extracts which displayed cytotoxic activities were sent to Harbor Branch Oceanographic Institution, Fort Pierce, Florida for cytotoxic assay utilizing a concentration of 5 μg/ml. The samples were tested in the pancreatic cancer cell line PANC‐1 and the multidrug‐resistant ovarian cancer cell line NCI/ADR (Table 3). The details of the cytotoxicity assay are described in an earlier publication (Gunasekera, Zuleta, Longley, Wright, & Pomponi, 2003). In brief, the cell lines were cultured in RPMI medium supplemented with 10% fetal bovine serum and were placed in a 5% CO_2_ incubator at 37°C. The antiproliferative effects of the test extracts were determined against the cell lines (200 μl cultures at 10^5^ cells/ml) in drug‐free medium or medium with the test agents at 5 μg/ml. All experimental cultures were initiated in medium containing gentamycin sulfate (50 μg/ml). After 96 hr exposure, cells lines were counted using 3‐[4,5‐dimethylthiazol‐2‐yl]‐2,5‐diphenyltetrazolium bromide (MTT).

### 2.4 Gram staining, nucleic acid extraction, 16S rRNA amplification, sequencing, and phylogenetic analysis

The Gram reaction of all pure cultures was determined via the nonstaining KOH method (Buck, 1982). The nucleic acid extraction involved the standard PCR isolation conditions. The reaction was boiled for 10 min (after the addition of nuclei lysis solution) to lyse the cells and the DNA pelleted by centrifugation at 13,000 rpm for 3 min. DNA concentration and purity were estimated using a nanodrop spectrophotometer. Absorbances at 230, 260, 280, and 320 nm were recorded. DNA purity was evaluated by dividing A260 by A280, with pure DNA having an A260/280 ratio of 1.8 (with a ratio of 1.4–2.0 being an acceptable working range). Samples were diluted using sterile water to achieve a DNA concentration within the range of 1–5 ng/μl for PCR processing.

PCR was conducted using the primers 27F (5′‐AGAGTTTGATCCTGGCTCAG‐3′) and 1492R (5′‐TACGGCTACCTTGTTACGACTT‐3′), targeting the 16S rRNA region of the domain Bacteria (Lane, 1991). The cycling conditions were: initial denaturation at 94°C for 1 min; 30 cycles of 94°C for 45 s, 55°C for 1 min, 72°C for 1.5 min with a final extension at 72°C for 10 min. Amplified 16S rRNA fragments were analyzed using agarose gel electrophoresis. The correctly sized PCR products were cut out of the gel with a sterilized scalpel and the DNA was purified from the agarose using a Qiagen's gel extraction kit assuming a gel weight of 200 mg.

DNA products were sequenced on ABI 3730XL capillary DNA sequencers at Sequetech Corporation in Mountain View, CA and at the Dana Farber/Harvard Cancer Center DNA Resource Core, Boston, MA. While all 16S rRNA fragments were partially sequenced using the 27F primer, the nearly complete 16S rRNA gene sequences of four isolates, 23MM, 38M1, 40M1, and 42M1, were obtained using with five additional sequencing primers: 1492R, 936R (5ʹ‐GTGCGGGCCCCCGTCAATT‐3ʹ), 519F (5′‐CAGCAGCCGCGGTAATAC‐3′), 519R (5ʹ‐GTATTACCGCGGCKGCTG‐3ʹ), and 1114F (5ʹ‐GCAACGAGCGCAACCC‐3ʹ).(Gontang et al., 2007; Mao, Zhou, Chen, & Quan, 2012).

All nucleotide sequences were assembled, analyzed, and manually edited using the Sequencher software package (version 4.8; Gene Codes Co., Ann Arbor, MI) and compared to sequences within the NCBI database (http://www.ncbi.nlm.nih.gov/) using the Basic Local Alignment Search Tool (BLAST). The partial 16S rRNA gene sequences of all isolates and their nearest type strain were analyzed using the phylogeny.fr ‘one click’ phylogenetic analysis tool (http://www.phylogeny.fr/version2_cgi/index.cgi) (Dereeper, Audic, Claverie, & Blanc, 2010; Dereeper et al., 2008). 16S rRNA gene sequences have been deposited in the GenBank database (http://www.ncbi.nlm.nih.gov/GenBank/index.html) under the accession numbers KM357365–KM357385.

## 3 Results

Sediments were collected from five locations off the Florida coast at depths ranging from 0.9 to 6 m (Table 1). A clump of the green‐grey sponge *H. panicea* (Hooper & van Soest, 2002; Little, 1963) was collected at a depth of 1.5 m. The sediment at depths of 3–6 m was comprised predominantly of sand and silt while sediments collected in shallower areas were sand covered with microalgae. The sediments were processed using previously established pretreatment methods outlined in Methods (Gontang et al., 2007).

**Table 1:** Sediment collection sites and isolation media of microbial isolates

Collection site (depth)	Strain isolated	Isolation media^a^
Florida Keys (0.3–0.6 m)		
	32NM	Nutrient, Seawater
Fort Pickens (4.3–6 m)		
	43NM	Nutrient, Seawater
	44NM	Nutrient, Seawater
Pensacola Bay (3–3.7 m)		
	39ND1	Nutrient, DI water
	34M1	M1 without rifampin
	33MD1	Marine, DI water
	25MD1	Marine, DI water
	23MM^b^	Marine, Seawater
	19MM^b^	Marine, Seawater
	27MD1	Marine, DI water
	42M1^b^	M1 without rifampin
	38M1^b^	M1
	35M3	M3
	36M3	M3
	40M1^b^	M1 without rifampin
Pensacola Beach (4.3–6 m)		
	47MM^b^	M1 and M3
Port St. Joe (sediment, 0.9–1.8 m)		
	12MD1^b^	Marine, DI water
	3MD1	Nutrient, DI water
Port St. Joe (sponge, 0.9–1.8 m)		
	BND1	Nutrient, DI water
	DMM^b^	Marine, Seawater
	MAR^b^	Nutrient, DI water

a The isolates were grown in liquid media similar to the agar isolation media.

b The cytotoxic potential of these isolates were tested.

Eighteen cultivatable morphotypes were picked from marine sediments and three from the sponge *H. panicea* (MAR, DMM and BND1) from areas off the Florida coast. We specifically employed isolation and growth conditions that would foster the isolation of spore‐forming Gram‐positive bacteria,(Gontang et al., 2007) the group of bacteria most commonly associated with the production of bioactive molecules.

### 3.1 Molecular taxonomy of microbial isolates

The 21 isolates obtained were selected for phylogenetic analysis based on their unique colors and gram stain morphologies. The phylogenetic analysis was accomplished using the NCBI nucleotide BLAST search and comparing the partial 16S rRNA gene sequences of the cultured strains with previously identified microorganisms. The isolates’ accession numbers (KM357365–KM357385) and their closest matches are listed in Table 2.

**Table 2:** List of isolates generated using a 16S rRNA sequence identity (%) to the nearest strain type

Phylogenetic Group (phylum/class)	Representative isolate (accession no.)	Sequence Length (bp)	Results of BLAST Analysis	
			Nearest Type Strain (accession no.)	Sequence Identity (%)
Actinobacteria				
	39ND1 (KM357376)	410	*Kocuria flava* (NR_044308)	98.8%
	12MD1^a^ (KM357367)	600	*Mycobacterium iranicum* (NR_117909)	99.0%
Alphaproteobacteria				
	32NM (KM357374)	665	*Erythrobacter flavus* (NR_025245)	100.0%
	34M1 (KM357379)	525	*Erythrobacter flavus* (NR_025245)	100.0%
	33MD1 (KM357370)	485	*Erythrobacter nanhaisediminis* (NR_116764)	99.0%
Bacteroidetes				
	BND1^b^ (KM357366)	780	*Algoriphagus chordae* (NR_025603)	97.3%
	DMM b ^,^ a (KM357385)	880	*Algoriphagus chordae* (NR_025603)	97.4%
Firmicutes				
	25MD1 (KM357375)	800	*Bacillus algicola* (AY228462)	99.5%
	47MM^a^ (KM357377)	310	*Bacillus flexus* (KJ569089)	99.0%
	23MM^a^ (KM357384)	1516	*Bacillus hwajinpoensis* (NR_025264)	99.8%
	19MM^a^ (KM357369)	220	*Bacillus vietnamensis* (NR_024808)	99.1%
	27MD1 (KM357371)	530	*Bacillus vietnamensis* (NR_024808)	99.2%
	42M1^a^ (KM357383)	1513	*Bacillus vietnamensis* (NR_024808)	98.7%
	43NM (KM357378)	600	*Bacillus vietnamensis* (NR_024808)	99.2%
	MAR b ^,^ a (KM357368)	855	*Brevibacillus formosus* (NR_040979)	99.8%
	44NM (KM357373)	525	*Sporosarcina koreensis* (NR_043526)	98.3%
Gammaproteobacteria				
	3MD1 (KM357365)	200	*Pseudomonas aeruginosa* (HE978271)	100.0%
	38M1^a^ (KM357381)	1500	*Pseudomonas balearica* (NR_025972)	99.7%
	35M3 (KM357380)	450	*Pseudomonas aestusnigri* (NR_126210)	99.3%
	36M3 (KM357372)	270	*Pseudomonas stutzeri* (AF094748)	100.0%
* *	40M1^a^ (KM357382)	1498	*Pseudomonas stutzeri* (AF094748)	99.7%

a The cytotoxic potential of these isolates were tested.

b The sponge microbial isolates.

**Figure 3:**
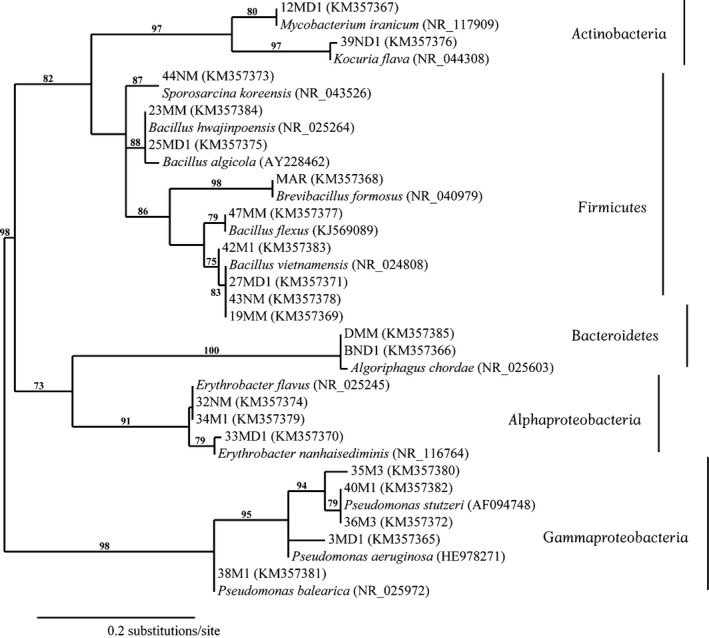
Neighbor‐joining distance tree based on the aligned 16S rRNA gene sequences of 21 cultured isolates and their nearest type strains. GenBank accession numbers are given in parentheses following the strain identification. Bootstrap values (in percent) are shown at the nodes for values greater than or equal to 60%

The results, based on partial 16S rRNA sequences**,** revealed the Gram‐negative *Pseudomonas* (from Pensacola Bay and Port St Joe) and Gram‐positive *Bacillus* (from Pensacola Bay, Pensacola Beach, Fort Pickens and Port St. Joe regions) as the two dominant genera (Figure 3). All of the sponge‐associated 16S rRNA sequences (MAR, DMM, and BND1) revealed greater than 97% sequence identity to the nearest type strain in the GenBank, while those from sediments mainly showed 16S rRNA gene sequence identities higher than 98%. Four isolates shared >99% 16S rRNA gene sequence identity with *Pseudomonas* type strains. Two of these four isolates shared 100% identity with the previously cultured *Pseudomonas* *stutzeri* or *P*. *aeruginosa*. The majority of the *Pseudomonas* strains were obtained from the Emanuel Point archeological shipwreck site in the Pensacola Bay area.

Of the *Bacillus* isolates, seven shared ≥99% 16S rRNA gene sequence identities with members of this taxon. The majority of the *Bacillus* sp. was obtained from the aforementioned archaeological shipwreck site in the Pensacola Bay area. Three *Erythrobacter* isolates shared ≥99% 16S rRNA gene sequence identity with their nearest type strains. Two strains that shared 100% gene sequence identity with the hitherto isolated *Erythrobacter flavus* strain that was collected from two different locations (Florida Keys and Pensacola Bay sediment collections).

A greater diversity of microbial isolates was noted from the Emanuel Point archaeological shipwreck site in the Pensacola Bay area compared to the other collection sites. The sediment‐associated bacterial community of this site was comprised of the phyla Actinobacteria, Firmicutes, and Proteobacteria (Alpha‐ and Gamma*‐* classes). In contrast, the diversity of the sponge *H. panicea*‐derived bacterial community from Port St. Joe area was limited to strains that were morphologically similar to the genera *Algoriphagus* and *Brevibacillus*. 16S rRNA gene sequence identities of >97% and >99% were observed for the strains related to members of the genera *Algoriphagus* and *Brevibacillus*, respectively. Two strains were isolated from the order Actinomycetales; one isolate from the Pensacola Bay area shared 99% 16S rRNA gene sequence identity with a *Kocuria*‐type strain, while the other, isolated from the Port St. Joe location, shared 98.8% 16S rRNA gene sequence identity with a *Mycobacterium*‐type strain. A 16S rRNA gene sequence identity of >98% with a strain from the genus *Sporosarcina* for was also noted for an isolate collected from the Fort Pickens area.

### 3.2 Seawater requirement of culturable bacteria

The majority of the microbial isolates were cultured on media containing seawater. The use of isolation media with and without seawater; however, did not appear to affect the isolation of certain *Pseudomonas*,* Bacillus*,* Erythrobacter*, and *Algoriphagus* genera. The *Sphingomonas* and *Sporosarcina* genera were isolated in seawater while *Mycobacterium* and *Kocuria* genera did not require seawater for growth.

The use of different isolation media with the same samples aided in the cultivation of diverse microbes. The marine and M3 isolation media were mainly selective for the *Pseudomonas* and *Bacillus* genera while a variety of other genera were obtained from other media. The use of nutrient‐poor versus nutrient‐rich media did not appear to alter the diversity of microorganisms isolated.

### 3.3 Biological activities of bacteria isolates

Of the cultivatable isolates, nine representative morphotypes from the various microbial classes were tested against the indicators: *P. aeruginosa*,* B. subtilis*,* S. aureus*,* C. albicans*, and *S. cerevisiae* (Table 3).

**Table 3:** Cytotoxic activities of selected microbial extracts

Extract	Strain	Location	Yeast^a^		Gram‐positive^a^		Gram‐negative^a^	Conc (5 μg/ml)^b^^,^ c % inhibition	
			*C. albicans*	*S. cerevisiae*	*S. aureus*	*B. subtilis*	*P. aeruginosa*	PANC‐1	NCI/ADR
23MM	*B. hwajinpoensis*	Pensacola Bay	+++	–	–	–	–	0	10
19MM	*B. vietnamensis*	Pensacola Bay	+++	–	–	–	–	9	18
42M1	*B. vietnamensis*	Pensacola Bay	–	–	+	++	–	18	18
38M1	*P. balearica*	Pensacola Bay	–	–	–	–	–	8	20
40M1	*P. stutzeri*	Pensacola Bay	–	–	++	++	–	18	26
47MM	*B. flexus*	Pensacola Beach	–	–	–	–	–	18	26
12MD1	*M. iranicum*	Port St. Joe	+++	–	–	–	–	0	16
DMM^d^	*A. chordae*	Port St. Joe	+++	–	++	++	–	32	20
MAR^d^	*B. formosus*	Port St. Joe	+++	–	–	–	–	4	16
NY	–	–	+++	+++	ND	ND	ND	ND	ND
GM	–	–	ND	ND	ND	+++	+++	ND	ND
CB	–	–	ND	ND	+++	ND	ND	ND	ND

a Activity is classified according to the diameter of the inhibition zone (+++ = ≥15 mm; ++ = 10–14 mm; + = ≤9 mm; – = no activity; ND = Not Done), NY = Nystatin (100 U), GM = Gentamicin (10 μg), CB = Carbenicillin (100 μg).

b Pancreatic cancer cell line (PANC‐1) and the multidrug‐resistant ovarian cancer cell line (NCI/ADR).

c No significant activity.

d The sponge microbial isolates.

The highest cytotoxic activities were observed from the extracts of microbes isolated from the shipwreck site. Strains 42M1 (*Bacillus vietnamensis*) and 40M1 (*Pseudomonas * *stutzeri*), for example, exhibited mild to moderate activities against the Gram‐positive *S. aureus* and *B. subtilis*, while extracts from strains 23MM (*Bacillus hwajinpoensis*) and 19MM (*B. vietnamensis*) both showed significant bioactivities against *C. albicans*. No cytotoxic activity was noted for the extract belonging to strain 38M1 (*Pseudomonas balearica*).

Cytotoxic activities were also seen for the extracts of microbes collected from the Port St. Joe area. The sponge‐derived microbial extract of strain DMM (*Algoriphagus chordae*), for instance, displayed moderate to significant activities against *C. albicans*,* S. aureus*, and *B. subtilis*. Good activities were also noted for the microbial extracts belonging to 12MDI (*Mycobacterium iranicum*) and MAR (*Brevibacillus formosus*). No significant cytotoxic activities were noted in the other antimicrobial assays nor against the mammalian pancreatic cancer (PANC‐1) and the multidrug‐resistant ovarian cancer (NCI/ADR) cell lines at an extract concentration of 5 μg/ml.

## 4 Discussion

This study utilized cultivation‐dependent methods to assess the diversity of microorganisms associated with marine sediments and the sponge *H. panicea* collected from Florida coastal waters. Overall, phylogenetic analyses revealed nine Bacillales, five Pseudomonadales, three Sphingomonadales, two Sphingobacteriales, and two Actinomycetales. *Pseudomonas* and *Bacillus* were the two dominant genera isolated from sediments associated with the archeological shipwreck site. Other isolates (i.e., *Erythrobacter*,* Algoriphagus*, and *Kocuria*) represented other genera that did not conform to the two aforementioned dominant genera. The limited number of actinomycetes obtained could have been due to the location and/or the shallow depth (0.9–6 m) from which the samples were collected (Jensen, Dwight, & Fenical, 1991), and future work will need to expand the range of cultivation conditions to determine if additional actinomycetes can be successfully isolated.

The extracts of microbes associated with the sponge *H. panicea* (MAR, DMM, and BND1) and those from the marine sediments displayed cytotoxic activities in the antimicrobial assays but not in the mammalian assays (Table 3). Microbial extracts that displayed antifungal activity were diminished in potency in their respective antibiotic assays. Antibiotic activities were only seen against the Gram‐positive bacteria. No significant bioactivity was observed against the Gram‐negative *P. aeruginosa*. These cytotoxic observations (especially the difference in activity between strains of the same genus‐23MM, 19MM, and 42M1) could reside in the fact that microorganisms, even those sharing the same 16S rRNA gene (Table 2), can have very different genomes (Dobrindt et al., 2003). It is possible that even if two strains share the same 16S rRNA gene, one may be missing the biosynthetic pathway or simply not be expressing the pathway needed for the production of a bioactive secondary metabolite.

**Figure 4:**
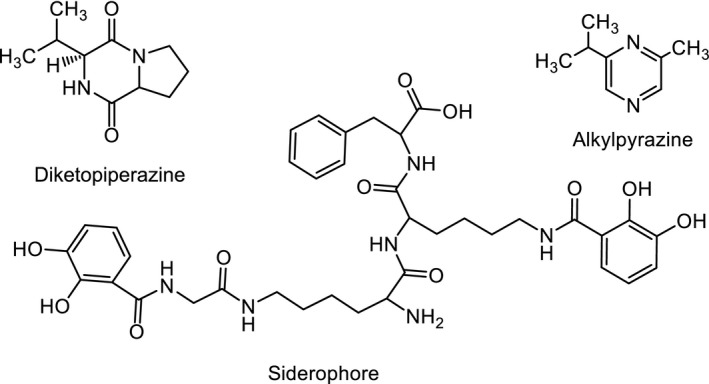
Reported chemical entities from other *P. stutzeri* strains

The observed bioactivities in our assays are in agreement with previous reports that confirms the potential of the above strains to facilitate the production of bioactive compounds(Bañeras et al., 2013; Gholizadeh, Bahador, & Baserisalehi, 2013; Phelan et al., 2012; Zawadzka, Vandecasteele, Crawford, & Paszczynski, 2006). One such example includes the isolation of chemical entities from other *P. stutzeri* species that have yielded compounds such as alkyl‐substituted diketopiperazines, pyrazines, and siderophores (Figure 4; Bañeras et al., 2013; Zawadzka et al., 2006). The diketoperazine that was isolated from the *P. stutzeri* strain ST1302 was shown to be active against the pathogenic fungus‐like *Pythium insidiosum* using the disk diffusion method (Thongsri et al., 2014). The *P. stutzeri* strain (40M1) which was active in our bioassay against both the Gram‐positive *S. aureus* and *B. subtilis* (Table 3) is likely to yield similar classes of bioactive compounds. It is also expected that the use of coculture experiments of with other microorganisms(Derewacz, Goodwin, McNees, McLean, & Bachmann, 2013) or the use of antibiotic challenge(Derewacz, Covington, McLean, & Bachmann, 2015) will lead to novel metabolomes.

The different sample collection sites revealed minor variation in their microbial diversity. More diversity was observed in areas with silty nutrient‐rich sediments (e.g., Pensacola Bay area) compared to sandy nutrient‐poor regions (e.g., Pensacola beach and Fort Pickens). These data could suggest that with greater diversity, the greater the potential for the production of potent secondary metabolites by the microbes (via quorum sensing to regulate the expression of virulence factors) for the sake of survival (Harder et al., 2014; Persson et al., 2009). In the case of the latter, the pathogenic *Bacillus flexus* appeared as the prevalent species and it exhibited cytotoxic activity in our assay. Its prevalence may be attributed to its ability to form endospores in order to survive under harsher nutrient poor conditions.

It is also worth noting the difference in the microbial community of the sponge‐associated microbes compared to its nearby sediment‐associated microbes from the same location in Port St. Joe (Table 1). The *Brevibacillus* sp. and *Algoriphagus* sp., for instance, were isolated from the sponge *H. panicea* while the *Mycobacterium* sp. and *Pseudomonas* sp. were obtained from sediments nearby the sponge. It is possible that these microbial specific locations can be correlated with their functional diversity in the marine ecosystem (Hooper et al., 2002; Lopanik, 2014; Proksch, 1994). The sponge‐associated microbes are able to evade the sponge's chemical defenses through symbiotic relationships while the nearby sediment‐associate microorganisms are unable to do this. The microbes are able to protect the sponge from other invaders through the production of their secondary metabolites(Perdicaris, 2013), the processing of metabolic waste compounds, the stabilization of the sponge skeleton and protection against UV radiation (Mehbub et al., 2014; Thomas et al., 2010).

Interestingly, isolates of the same genera and species were located at different collection sites. The *Erythrobacter flavus* species, for example, were both present at the Pensacola Bay and Florida Keys locations (Figure 2), and the *B. vietnamensis* species were collected at both Fort Pickens and Pensacola Bay. Since the Florida current is a well‐defined component of the Gulf Stream system (Johns & Schott, 1987), it is quite likely responsible for the distribution of these microorganisms.

In conclusion, this study assessed the microbial communities associated with marine sediments and the sponge *H. panicea* from diverse and previously unexplored areas located off the Florida coast. Phylogenetic analyses of the isolated strains revealed the dominant groups as Proteobacteria, Firmicutes, Bacteroidetes, and Actinobacteria. Moreover, seven of the microbial isolates from the aforementioned phyla were shown as sources of promising antimicrobial agents. Future studies will examine the isolation and mechanism of action of these and potentially new cytotoxic agents from marine environs.

## Conflict of Interest

No conflict of interest is declared.
